# Supplementary material for: Spatio-temporal characterization of fracture healing patterns and assessment of biomaterials by time-lapsed in vivo micro-computed tomography
Source: Sci Rep. 2021 Apr 21;11:8660. doi: 10.1038/s41598-021-87788-6 (PMC8060377; doi:10.1038/s41598-021-87788-6)
Supplement: Supplementary file 1 — Supplementary Information 1. [file 41598_2021_87788_MOESM1_ESM.pdf]

Spatio-temporal characterization of fracture healing patterns and assessment of biomaterials by time-lapsed *in vivo* micro-computed tomography

Esther Wehrle[1], Duncan C Tourolle né Betts[1], Gisela A Kuhn[1], Erica Floreani[1], Malavika H Nambiar[1], Bryant J Schroeder[1], Sandra Hofmann[1,2], Ralph Müller[1]

[1] Institute for Biomechanics, ETH Zurich, Zurich, Switzerland, [2] Department of Biomedical Engineering and Institute for Complex Molecular Systems, Eindhoven University of Technology, The Netherlands.

Corresponding author:

Ralph Müller, PhD

Institute for Biomechanics

ETH Zurich

Leopold-Ruzicka-Weg 4

8093 Zurich, Switzerland

Email: ram@ethz.ch

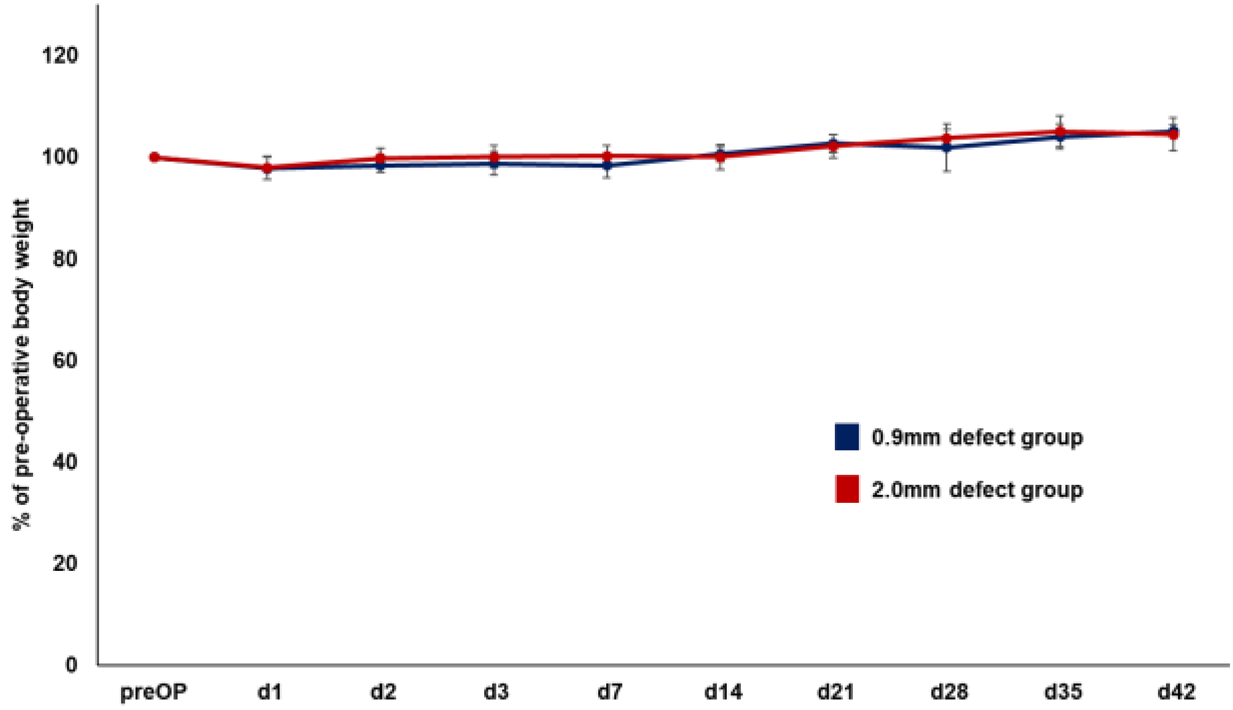

Supplementary Fig. S1. *In vivo* monitoring of body weight of the mice from the 0.9mm defect group (n=8) and the 2.0mm defect group (n=10) measured pre-operatively (preOP), on postoperative days 1-3 and weekly from day 7 to day 42. The postoperative values were related to the preoperative data.

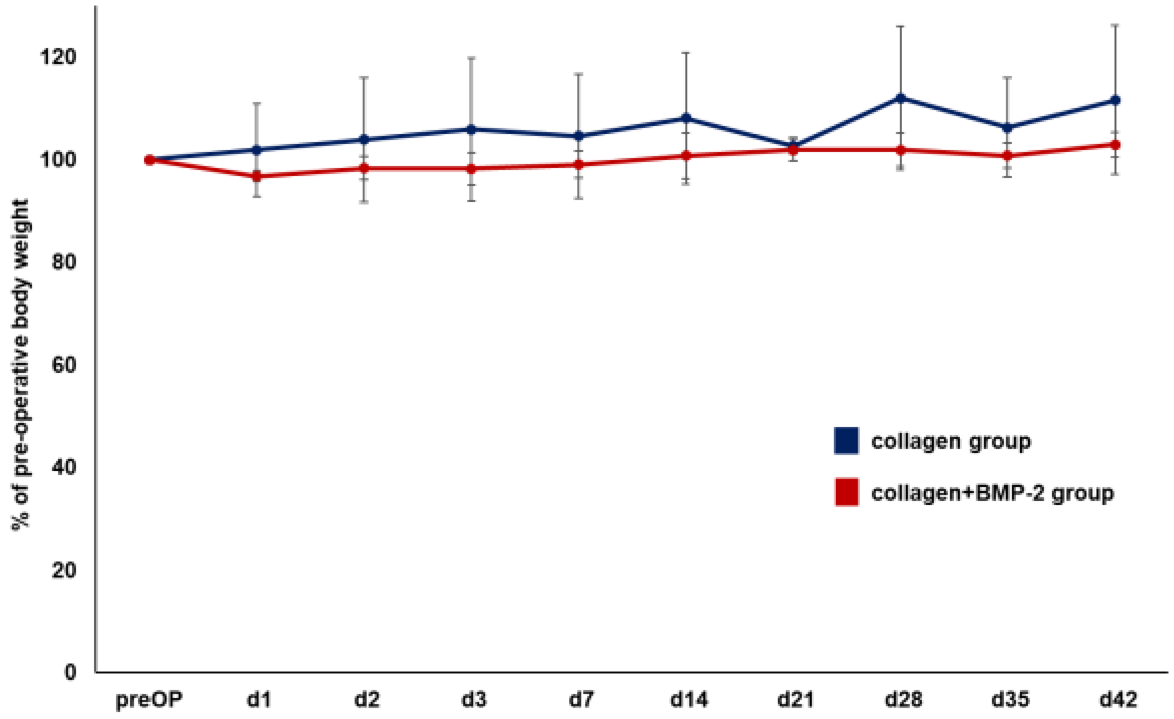

Supplementary Fig. S2. *In vivo* monitoring of body weight of the mice from the collagen group (n=8) and the collagen+BMP-2 group (n=8) measured pre-operatively (preOP), on postoperative days 1-3 and weekly from day 7 to day 42. The postoperative values were related to the preoperative data.

Supplementary Table S1. Study design (female 20 week-old C57BL/6J mice)

Experiment	Group	Group size	Size of femur defect	Biomaterial application	*In vivo* micro-CT measurements	Registration of micro-CT scans #	Histology
1	0.9mm	n=11	0.9mm (n=10)	-	d0, week 1-6 (n=10)	week 1-6 to week 0-5 (n=10)	week 6 (n=2)
	2.0mm	n=8	2.0mm (n=8)	-	d0, week 1-6 (n=8)	week 1-6 to week 0-5 (n=7)	week 6 (n=2)
2	collagen	n=8	2mm (n=8)	collagen	d0, week 5+6 (n=8)	week 1-6 to week 0-5 (n=8)	week 6 (n=1)
	collagen +BMP-2	n=8	2mm (n=8)	collagen +BMP-2	d0, week 5+6 (n=8)	week 1-6 to week 0-5 (n=8)	week 6 (n=1)

micro-CT scan taken at timepoint x registered to micro-CT scan taken at timepoint x-1

Supplementary Video 1. Visualisation of the defect healing process and the VOIs involved for a representative animal from the 0.9mm defect group and the 2.0mm defect group.

Supplementary Video 2. Visualisation of the defect healing process and the VOIs involved for a representative animal from the collagen group and the collagen+BMP-2 group.
